# Amazing Results with Hydroxyurea Therapy in Chronic Hepatitis B: A Preliminary Report

**Published:** 2010-09-01

**Authors:** Hessam Hassanzadeh Kashani, Aliakbar Vossoughi, Peyman Adibi, Seyed Moayed Alavian

**Affiliations:** 1 Department of Gastroenterology and Liver Diseases, AJA University of Medical Sciences, Tehran, Iran; 2 Gastroenterology Section, Department of Medicine Isfahan University of Medical Sciences, Isfahan, Iran; 3 Baqiyatallah Research Center for Gastroenterology and Liver Disease Baqiyatallah University of Medical Sciences, Tehran, Iran

**Keywords:** HBV, Chronic Hepatitis B, Hydroxyurea, Antiviral Therapy

## Abstract

**Background and Aims:**

To introduce the possible role of hydroxyurea (HU), a well-characterized antineoplastic drug with established antiviral effects, in the treatment of chronic hepatitis B.

**Methods:**

Four antiretroviral-naïve patients with chronic hepatitis B were enrolled in this limited pilot trial, and given 1000 mg/day of hydroxyurea for 4 weeks; then, the administration of the drug was suspended for 4 weeks. A clinical study and laboratory safety assessments and measurements of viral load were made at baseline, after drug therapy, and after one month suspension of the treatment

**Results:**

All 4 patients showed a significant decrease in viral load after 4 weeks of hydroxyurea therapy and the viral load of 2 patients increased again after a 4-week suspension of hydroxyurea

**Conclusions:**

Our data demonstrate that the old low-cost antineoplastic drug, hydroxyurea, efficiently blocks hepatitis B virus (HBV) replication. We suggest that HU will play an important role in the treatment of chronic hepatitis in the fore- seeable future. Further studies including those that evaluate optimal dosing in long-term use will continue to define the role of HU in the treatment of HBV infection alone or in combination with other antiviral drugs

## Introduction

Hepatitis B virus (HBV) has affected more than 2 billion people worldwide (about one third of the world's population) with more than 350 million people chronically infected. Chronic hepatitis B is a dangerous disease that can lead to liver cirrhosis and subsequently to hepatocellular carcinoma (HCC)[1].

HBV is a double-stranded DNA virus belonging to the hepadnavirus family replicating an intermediate RNA, the pregenomic RNA (pgRNA); this very much resembles the mechanism of HIV virus replication[1][2].

Hydroxyurea (HU) is a drug with a history of more than 40 years use in the treatment of hematological malignancies such as chronic myeloid leukemia (CML) and diseases like sickle cell anemia, polycythemia vera, and psoriasis. HU is a free radical quencher, and inhibits the cellular enzyme ribonuclotide reductase, the rate limiting enzyme responsible for the conversion of ribonucleotides to deoxyribonucleotides, which is essential for DNA synthesis (3). This amazing biological effect has stimulated interest in HU for the treatment of diseases which are mediated by DNA viruses such as human immunodeficiency virus (HIV), herpes simplex virus (HSV) and polyomavirus[4][5][6].

This astonishing characteristic of HU has marked the last decade for its initiation of the application of HU to AIDS therapy, for the first time in history[7].

Since there is abundant evidence for the positive effects of HU in decreasing HIV and HSV replication, and because of the similarity of the HIV and HBV replication processes, we have postulated HU's possible effect in decreasing HBV replication.

## Materials and Methods

To explore whether HU could be used to decrease HBV viral load, we conducted a limited trial on patients with chronic hepatitis B.

The four patients whose chronic hepatitis B positivity was definite were male with an age range of 29-58. They had not received any kind of treatment before they participated in our research. None of them had any sign of being previously or presently affected by hepatitis A, C, D, and HIV. Liver biopsy also indicated their active chronic hepatitis with HBV. Other routine tests including complete blood count (CBC), albumin and prothrombin time showed completely normal results.

All patients were fully informed of the effects of the drug and instructed in its use. Afterwards, they gave their informed consent so as to be allowed to participate in the study. For four weeks on a daily basis, the patients received 1000 mg HU medication. During this four-week therapy period, the patients were examined once a week for any possible side effects and underwent a CBC test. We evaluated the HBV replication rate by estimating HBV viral load before and after the first month of treatment with HU, by means of the polymerase chain reaction (PCR), similar to the method followed in previous studies [8]. In order to confirm the results, all patients were retested for HBV viral load one month after the end of the treatment.

The levels of viral load prior to the study were more than 106 copies/mL. Each patient was given this test three times: prior to the treatment, at the end of drug therapy, and one month after the end of therapy.

## Results

Finally, after two months, none of the patients showed any side effects; on the contrary, wonderful effects of the drug were revealed. Complete viral load results during the study appear inTable 1andFig 1

**Fig 1:**
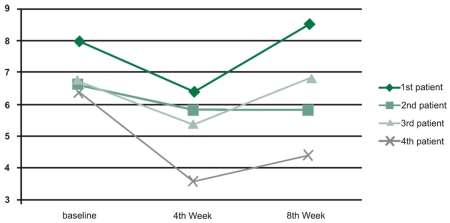
Kinetics of HBV during study (logarithmic).

**Table 1:** Dynamic parameters related to HBV viral load (copies/mL) of patients

	pat1 stients	2nd patient	3rd patient	4th patient
Baseline	95.55(mu)^a^106	4.47(mu)^a^106	5.89(mu)^a^106	2.62(mu)^a^106
After drug treatment	21.32(mu)^a^106	0.63(mu)^a^106	2.49(mu)^a^106	0.0
After drug suspension	314(mu)^a^106	0.67(mu)^a^106	6.84(mu)^a^106	0.02(mu)^a^106

^a^ Multiple

## Discussion

As mentioned before, the most important goal in the treatment of chronic hepatitis B is to reduce HBV load; and HU was shown to be capable of putting this into practice. Moreover, a recurring increase in HBV in half of the patients (two out of four) after a month of drug suspension strengthens our conclusions. Finally, given the HBV load of zero in one of the patients, the hypothesis of the HU effect in hepatitis B treatment is further confirmed.

The findings of the present study are to be considered as the primary basis upon which further comprehensive and more detailed studies are recommended for the evaluation of the impact of HU as a possible drug effective in the treatment of hepatitis B. HU is simply absorbed orally and any probable or rare side effects, in case of occurrence, can be prevented by discontinuation of the drug [3][7].

It is worth mentioning that any claim that a certain drug is capable of fully hindering HBV replication should be accompanied by its practical effect on the reduction of initial viral load by more than 2 logs [8]; this happened in the case of just one of our patients. Furthermore, the very short HU treatment time span of the present study should not be overlooked. While most of the drugs administered to target hepatitis B take four months for their effects to appear, HU manifested its wonderful effectiveness after less than a month. Therefore, we anticipate even greater effects of HU in suppressing HBV replication, the longer the duration of treatment

Altogether, there are reasons to support the claim that HU is a very excellent drug for the treatment of hepatitis B:

1.HU has been a well-recognized drug throughout its years of use.

2.It is simply absorbed orally and is easily absorbed into all bodily fluids.

3.Toxicity of this drug is very low and in case of any side effects mere discontinuation can end complications

4.The most important reason for drug resistance is mutation. HU, by causing a reduction in the production and replication of HBV in fact lowers the chance of mutations, thereby reducing resistance.

In any case, it seems that further and more comprehensive studies of HU in isolation or in combination with other drugs can be very helpful in shedding light on all relevant aspects.
